# Anterior and posterior uveitis associated with juvenile idiopathic arthritis -case report

**DOI:** 10.22336/rjo.2022.36

**Published:** 2022

**Authors:** Lorina Petrescu, Mirela Crișan, Călin Lazăr, Cristina Stan

**Affiliations:** * Department of Ophthalmology “Iuliu Hațieganu” University of Medicine and Pharmacy Cluj-Napoca, Romania; Department of Ophthalmology, County Emergency Hospital, Cluj-Napoca, Romania; ** Pediatrics I Department, Clinical Emergency Hospital for Children, Cluj-Napoca, Romania; *** “Iuliu Hațieganu” University of Medicine and Pharmacy Cluj-Napoca; Pediatrics I Department, Clinical Emergency Hospital for Children, Cluj-Napoca, Romania

**Keywords:** uveitis, juvenile idiopathic arthritis, chorioretinitis, COVID-19

## Abstract

Anterior uveitis is the most common extra-articular manifestation in children diagnosed with Juvenile idiopathic arthritis (JIA).

It is typically a non-granulomatous, chronic, and asymptomatic uveitis. The lack of acute symptoms often delays the diagnosis with the incidence of severe ocular complications. Chorioretinitis lesions have been described in only 1% of cases. The absence of fundus changes can be explained by the impossibility of performing fundoscopy through the cloudy ocular media, secondary to inflammation.

A 7-year-old female with a 3-month history of painless reduced vision came to have an eye examination. An initial diagnosis of bilateral anterior granulomatous uveitis complicated with glaucoma and cataract was formulated. Because of the concomitant diagnosis of COVID-19 disease (same day as the eye examination), the child was hospitalized in a hometown COVID-19 patient ward, so both local and general treatment, monitorization, and investigations were discontinued. The following eye examination revealed the persistence of anterior uveitis, inflammatory glaucoma, cataract, and the appearance of band keratopathy. Fundoscopy revealed numerous disseminated lesions of choroiditis. Further examinations established JIA-associated uveitis diagnosis, so systemic corticosteroids were initiated followed by Methotrexate and Adalimumab.

**Monitoring with fundoscopy in a patient diagnosed with JIA-U is necessary to detect possible chorioretinal or vascular damage**.

**Abbreviations:** BVA = best visual acuity, CVA = corrected visual acuity, CS = corticosteroids, IOP = Intraocular pressure, JIA = Juvenile idiopathic arthritis, JIA-U = Juvenile idiopathic arthritis associated uveitis, LE = left eye, MTX = Methotrexate, OU = both eyes, OCT = Optical Coherence Tomography, RE = right eye, TNF = tumor necrosis factor

## Introduction

Childhood uveitis accounts for 5-10% of all cases of uveitis. One of the most common causes of pediatric non-infectious uveitis is JIA [**1**].

JIA is an autoimmune condition that affects children younger than 16. Anterior non-granulomatous uveitis is the most common extra-articular manifestation of JIA and may precede or appear after the onset of arthritis [**2**,**3**]. It is characterized by silent progression (no ocular pain or ciliary injection), chronic evolution, and complications that can lead to severe vision loss (cataract, glaucoma, band keratopathy). The first-step treatment of JIA-associated uveitis (JIA-U) includes local and general corticosteroids (CS) and mydriatics and the following steps consist in Methotrexate and biologic therapy (anti-TNF-alpha). Surgical treatment of eye complications is a real problem due to the persistence of intraocular inflammation [**4**].

Because of the reorientation of the health system towards patients related to COVID-19, traffic restrictions imposed by the authorities, or fear of COVID-19 infection among the population, the access of patients with other pathologies to health services has been acutely restricted. As a result of this situation, many patients experienced the worsening of pre-existing diseases or the appearance of new conditions that remained undiagnosed until late.

## Case report

A 7-year-old female with a 3-month history of painless reduced vision was brought to an eye consultation by her relatives in June 2020.

The delay of the consultation for three months had some reasons: the implementation of travel restrictions during the coronavirus pandemic with no possibility of leaving the hometown, and probably the lack of eye pain did not alert the relatives. 

The examination revealed the following: Right eye BVA = 1/ 50, Left eye BVA = 5/ 50 (Snellen).

**Fig. 1:**
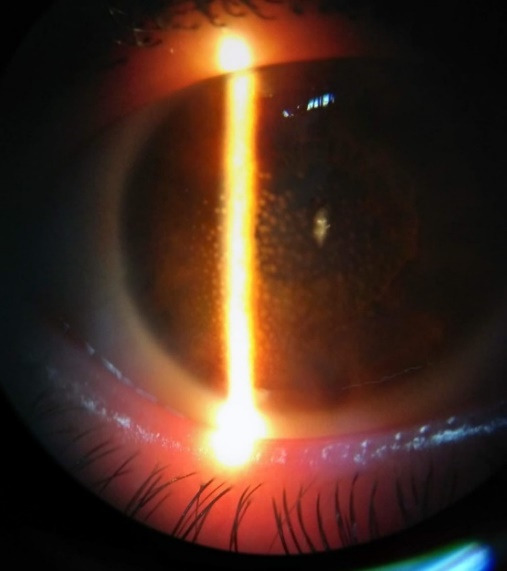
Large “mutton fat” keratic precipitates

Intraocular pressure (IOP) = high on digital palpation. Biomicroscopy of both eyes: large “mutton fat” keratic precipitates, presence of cell in the anterior chamber, iris nodules, lens opacities (**Fig. 1**). Anterior pole changes provided no view of the fundus. 

**The initial diagnosis was: bilateral granulomatous acute anterior uveitis complicated with glaucoma and cataract.**

Therefore, the patient was urgently hospitalized for investigations and treatment: systemic CS + proton pump inhibitor, local anti-inflammatory, mydriatic, and antiglaucoma agents.

Laboratory test results: ESR, CRP - within normal limits; ASLO, VDRL, TPHA, Borrelia Ab (IgM, IgG), T. Gondii Ab (IgM, IgG), CMV Ab, Herpes 2 Ab (IgM, IgG), HAV Ab (IgM, IgG), MPO Ab, PR-3 Ab - were all negative. 

Because of the concomitant diagnosis of COVID-19 disease (same day as the ophthalmological examination), the child was admitted to a hometown hospital with a COVID-19 patient ward, with recommendations for local and general treatment and completing the investigations to establish the etiology.

Between June 2020 and January 2021, the patient did not have any eye examination and did not follow any treatment. 

In March 2021, the patient returned to our clinic. The slit-lamp examination revealed: bilateral persistent anterior granulomatous uveitis with the aggravation of the existing complications and the debut of band keratopathy.

BVA-OU = 2/ 50;

Intraocular pressure: RE = 54 mmHg, LE = 45 mmHg (Goldman aplanotonometry).

**Fig. 2:**
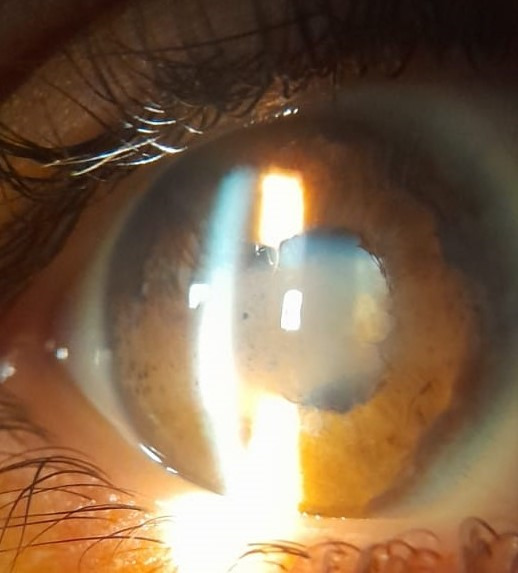
Band-like interpalpebral calcification and posterior synechiae

Anterior segment examination showed band-like interpalpebral calcification, “mutton fat” keratic precipitates, cell in anterior chamber SUN2+, iris nodules, lens opacities, anterior and posterior synechiae, seclusio, and occlusion pupillae. It was impossible to perform the fundoscopy (**Fig. 2**).

Except for COVID infection (June 2020), APP and AHC did not reveal any systemic disease.

Ocular ultrasound (US) showed no abnormalities.

**Diagnosis: Bilateral granulomatous chronic anterior uveitis, inflammatory glaucoma, complicated cataract, band keratopathy, and presumed JIA.**

The patient was urgently hospitalized for investigations and treatment with immunosuppressive doses of systemic steroids. She also received local treatment with anti-inflammatory, mydriatic, and antiglaucoma agents.

Due to suspicion of JIA-U, a pediatric examination was required, which was supplemented by paraclinical investigations that supported the diagnosis (ANA = positive, HLA-B27 positive). QuantiFERON-TB was negative and chest X-ray was normal. What should be mentioned is the positivity of anti-T. Gondii Ab, respectively anti CMV Ab, but these were negative in June 2020, when the eye disease was present and severe. 

Therefore, the patient was started on general treatment with Methotrexate and Adalimumab under pediatric follow-up.

Despite the maximum tolerated topical antiglaucoma therapy, the presence of high values of IOP required glaucoma surgery - bilateral trabeculectomy (March 2021), with favorable postoperative evolution: normal IOP on digital palpation. One week after, the patient underwent uncomplicated extracapsular cataract extraction with posterior chamber lens implantation (*ECCE+PC-IOL*) in the left eye. 

Between April and December 2021, the patient followed Methotrexate + Adalimumab therapy without systemic CS and came to regular 4-6 weekly eye follow-ups. Topical treatment (IOP lowering agents and minimal doses of CS depending on the degree of ocular inflammation) was also continued. Despite the general therapy with Mtx + Adalimumab, no suppression was observed, but only a reduction of inflammation.

BVA-OU: variations depending on the degree of eye inflammation (between 10/ 50-35/ 50).

**Fig. 3:**
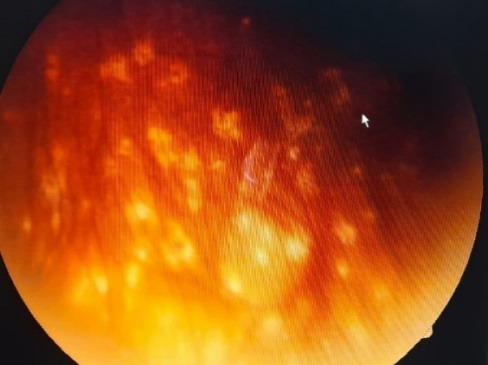
Fundus view with multiple hypopigmented spots

IOP was within normal limits (17-19 mmHg) under the topical IOP lowering agents. The reduction of anterior chamber inflammation in both eyes and cataract surgery performed in LE allowed fundoscopy through the peripheral cornea. Multiple hypopigmented spots were detected on the peripheral retina, but there was no perilesional hyperpigmentation or vitreous inflammation in the front of the lesions (**Fig. 3**).

**Fig. 4:**
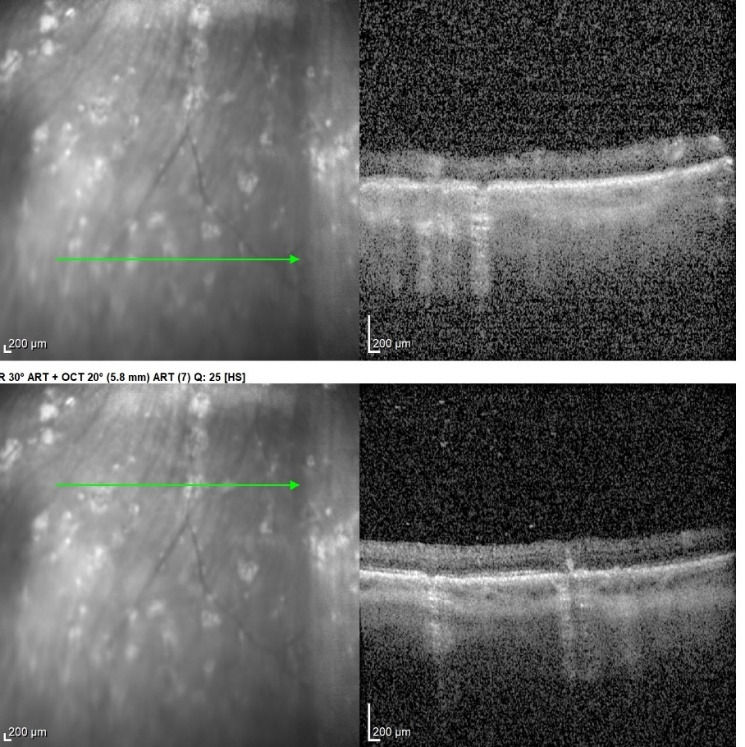
OCT - Destructions of the retinal pigment epithelium

Destructions of retinal pigment epithelial corresponding to the lesions found on fundoscopy were documented on OCT (Optical Coherence Tomography) (**Fig. 4**). No macular oedema was observed.

Given the progressive decrease in VA of the RE, cataract surgery was scheduled. In addition to Methotrexate + Adalimumab therapy and topical treatment: anti-inflammatory and IOP lowering agents, we decided to initiate a two-week general CS therapy before surgery. Hence, the ocular inflammation was significantly reduced and the *ECCE+PC-IOL* cataract surgery was performed with favorable postoperative evolution. At the postoperative consultation, an increase in the number of hypopigmented lesions was observed compared to the previous consultation (3 months before).

In January 2022, the patient’s eye condition was much improved:

RE CVA = 35/ 50-40/ 50 (-1.00 x 9);

LE CVA = 35/ 50-40/ 50 (-1.00, -1.00 x 180);

IOP OU = 18 mmHg (under IOP lowering agents).

Bilateral findings on biomicroscopy: superior conjunctival filtering bleb, band keratopathy, much more transparent cornea, few fine keratic precipitates, superior iris coloboma, IOL-pc.

Bilateral fundoscopy: multiple hypopigmented lesions without vitreous inflammation scattered all over the peripheral retina.

With MTX + Adalimumab therapy, the inflammatory process was only reduced, but through the introduction of the general CS, the evolution was noticeably improved.

**Diagnosis: Bilateral chronic granulomatous anterior uveitis, inflammatory glaucoma, complicated cataract, band keratopathy, disseminated multifocal choroiditis, JIA.**

**Differential diagnosis: infectious/ non-infectious?**

A. Infectious etiology

The laboratory investigations ruled out an infectious etiology. Additionally, the clinical features of the anterior uveitis sustained a non-infectious type because the former had acute symptoms (pain, photophobia).

The chorioretinal lesions observed postoperatively were numerous, unpigmented, with well-defined borders disseminated on the peripheral retina, and without vitreous inflammation, thus, toxoplasma was excluded as a cause (although anti-T. Gondii IgM and IgG were positive in March 2021). Also, a positive test for anti-CMV antibodies (positive in March 2021) in an immunocompetent patient does not support the CMV etiology of chorioretinitis.

B. Non-infectious etiology

1. The most common non-infectious cause of anterior uveitis in children is JIA [**1**]. In our patient, the diagnosis of JIA-U was sustained ophthalmologically: bilateral uveitis with “white” eyes and no eye pain, and pediatric: early-onset (7 years), positive ANA, and positive HLA B27.

JIA-U is bilateral in 70-80% of cases [**3**,**5**]. Typically, arthritis precedes the onset of uveitis with an average interval of 1.8 to 2 years [**6**,**7**]. However, in approximately 10% of cases, uveitis may precede the diagnosis of arthritis, and because of its asymptomatic course, the risk of ocular complications is significantly increased [**3**,**8**-**10**].

Asymptomatic chronic anterior uveitis is typical for JIA, but sometimes, especially in enthesitis-related JIA, anterior uveitis may be acute and symptomatic. In rare cases, progression to panuveitis is possible [**11**]. Some risk factors have been identified in JIA-U: female gender, oligoarticular type of JIA, younger age at onset, and AAN-positive or HLA-B27-positive.

2. Other causes of uveitis 

Juvenile spondyloarthropathy (JOSpAs) may associate anterior uveitis, but this is usually unilateral with congested, painful eyes and positive family history [**1**].

Juvenile sarcoidosis/ Blau syndrome: both conditions may develop with joint damage, granulomatous uveitis, and posterior pole lesions (chorioretinal lesions, which are atypical for JIA-U), but were missing: very young age of onset, cutaneous granulomatous lesions, radiographic pulmonary lesions. 

TINU (uveitis associated with tubulointerstitial nephritis) was excluded due to the lack of renal failure (negative laboratory tests).

Inflammatory bowel disease: no characteristic symptoms were observed.

Kawasaki disease, Behcet disease, Henoch-Schonlein purpura: vasculitis was in the foreground of diagnosis.

Masquerade syndromes in children that can present with uveitis include leukemia, retinoblastoma, JXG, and trauma. These causes were ruled out, based on laboratory examinations, ophthalmological examination, eye ultrasound and anamnesis [**12**].

The pediatrician’s final diagnosis was juvenile idiopathic arthritis.

## Discussions. Particularities of the case

1. Granulomatous anterior uveitis. JIA-U is typical non-granulomatous, but granulomatous forms can also exist [**13**,**14**]. The case presented had large keratic, precipitates iris nodules on the edge of the pupil, which placed it in granulomatous forms. Although the ocular inflammation was particularly intense in both the first and second presentation, no ciliary injection, pain, or photophobia was observed. The silent evolution of anterior uveitis in JIA and the possibility of the onset of uveitis before arthritis are well known [**9**].

2. Chorioretinitis lesions. Partial opacification of the ocular media, as a result of inflammation or as an expression of complications (cataract, band keratopathy) or simply due to the lack of cooperation of the patient (child), may impair the detection of the chorioretinal lesions on fundoscopy. 

Posterior segment complications include macular edema and epiretinal membrane. Chorioretinal lesions such as chorioretinitis, or placoid epitheliopathy, are cited in only 1% of cases of JIA-U [**15**-**17**]. 

In the present case, multiple chorioretinal depigmented lesions were found scattered on the peripheral retina. No perilesional hyperpigmentation and no vitreous inflammation in the front of the foci was observed. OCT showed retinal pigmentary epithelium destruction corresponding to the fundus lesions.

3. COVID-19 pandemic, delay in diagnosis and treatment.

The first medical visit only three months after the onset of the vision loss, interruption of the treatment, and the delay of the medical investigations were due to the restrictions and fears of the COVID-19 pandemic context.

The progression of JIA-U to sight-threatening complications is well known [**18**].

Our patient developed almost all the complications cited in literature: band keratopathy, cataracts, and inflammatory glaucoma. OCT showed no macular edema, instead, she developed chorioretinitis lesions.

## Conclusions

Decreased visual acuity in children, even without other symptoms, requires a complete eye examination.

Monitoring with fundoscopy in a patient diagnosed with JIA-U is necessary to detect possible chorioretinal or vascular damage. Choroiditis lesions may not be so uncommon in JIA-U.

**Conflict of Interest Statement**

The authors state no conflict of interest. 

**Informed Consent and Human and Animal Rights statement**

Informed consent has been obtained from the legal guardian of the patient included in the study.

**Authorization for the use of human subjects**

Ethical approval: The research related to human use complies with all the relevant national regulations, institutional policies, it is in accordance with the tenets of the Helsinki Declaration and has been approved by the review board of Department of Ophthalmology, County Emergency Hospital, Cluj-Napoca, Romania. 

**Acknowledgements**

None. 

**Sources of Funding**

None. 

**Disclosures**

None. 
